# Inferential collective self-knowledge

**DOI:** 10.1007/s44204-025-00324-5

**Published:** 2025-09-17

**Authors:** Lukas Schwengerer

**Affiliations:** 1 Institut für Philosophie, University of Graz, Heinrichstraße 26, Graz, Graz 8010 Austria; 2 Fakultät für Geisteswissenschaften, Institut für Philosophie, University of Duisburg-Essen, Universitätsstr. 12, Essen, 45141 Germany

**Keywords:** Collective self-knowledge, Group self-knowledge, Self-knowledge, Groups, Inferential self-knowledge

## Abstract

I develop an inferential account of collective self-knowledge. Starting with the assumption that groups have (at least propositional) attitudes I look at desiderata for any account of collective self-knowledge of such attitudes. Any such account has to explain the features that group avowals have in our ordinary linguistic practice. Moreover, any account ought to be compatible with as many views of group attitudes as possible. I propose a new account that looks at attitude-forming processes of groups as evidence for collective self-knowledge. It is based on forward-looking inferences in contrast to the backwards-looking conception commonly found in inferential accounts of self-knowledge in individuals. I then show that groups have a minimal form of privileged and peculiar access to their own attitudes because they have easy access to their own production of attitudes as evidence.

## Introduction

It is a widespread—although not uncontested—claim that groups can have propositional and non-propositional^1^ attitudes (Searle (1995, 2010), Tuomela (2004, 2005, 2013), Gilbert (1989, 1990; 2009; 2014), Pettit (2001, 2007), List (2005), List and Pettit (2011), Schmid (2009), Epstein (2015), Hawley (2017), Lackey (2021)). Groups can intend, want, or believe. Assuming that claim is correct, I ask how groups can know of their own attitudes. That there is collective self-knowledge has been argued for by Schwengerer (2022) and further discussed by Peterson (2022). But in this emerging area a proper account of how exactly groups can know their own attitudes has not been given yet. I aim to provide such an account.

I start by setting up three desiderata my theory of collective self-knowledge ought to satisfy. Two of those are about the nature of group avowals that have to be explained by a theory of collective self-knowledge. The third is a general constraint that the theory ought to be compatible with as many different background views on group attitudes as possible. The landscape of theories of group attitudes is anything but settled, hence it is a virtue if the account developed is not limited to a single of those accounts of group attitudes.

After determining the desiderata, I build a new account of collective self-knowledge based on forward-looking inferences. I illustrate it with two easily compatible background views by Gilbert and List & Pettit. I show how the account satisfies the desiderata for these relatively easy background views, before I finally take on the challenge of combining my proposal with an interpretationist view of attitudes that seems more difficult to combine with my approach.

## Desiderata

### Self-knowledge

Before developing the account of inferential collective self-knowledge, I need to lay out what exactly any such account ought to explain. The account aims to capture features that collective self-knowledge in general has. I am not establishing these features in this paper. Rather, I take them from Schwengerer (2022) where the features are argued for at length.

Schwengerer (2022) draws on Crispin Wright’s (1998, 2015) analysis of our ordinary linguistic practice in self-ascribing attitudes as individuals. Wright looks (among other things) at the paradigmatic speech act of self-ascribing an attitude^2^ and tries to establish desiderata for accounts of self-knowledge by looking at these speech acts. This speech act, labelled as an *avowal*, has particular features that any account of self-knowledge ought to explain. I will limit myself to avowals regarding attitudes to establish desiderata for collective self-knowledge. The reason for limiting myself to attitudinal avowals is that, even among friends of group attitudes, it is controversial whether groups have any phenomenal states and such states provide the paradigmatic examples of non-attitudinal avowals. There are some limited proposals for shared emotions that are—or at least come close to being—applicable to groups, but the majority remain skeptical (cf. León et al. (2019)). Hence, I limit myself to propositional attitudes and attitudinal avowals.

For avowals by individuals, Wright identifies three features, only two of which also have analog features in group avowals. Those two are as follows:

i) Weak authority: “attitudinal avowals would appear to exhibit a form of weak authority; nevertheless, that is, they provide criterial—empirically assumptionless—justification for the corresponding third‐person claims. Wholesale suspicion about my attitudinal avowals—where it is not a doubt about sincerity or understanding—jars with conceiving of me as an intentional subject at all. (Wright, 1998, pp. 17–18).
ii) Salience (originally “*transparency*”): “except where the matter is one of interpretation, we think a subject ought to know without further ado what she believes, or desires, etc., so that any profession of ignorance or uncertainty, unless coupled with a readiness to allow that the matter is not basic but calls for (self‐) interpretation, will seem perplexing” (Wright, 1998, pp. 16–17).

For group avowals, I work with the following—related but in some respects different—features based on Schwengerer (2022) for paradigmatic group avowals:

1. Weak authority: sincere attitudinal avowals provide empirically assumptionless justification for the corresponding third‐person claims. One can (even though one rarely does) doubt attitudinal avowals without doubting sincerity or understanding, if one has very good reasons to do so. But in ordinary circumstances, a challenge will be about sincerity or linguistic competence, not about the group being mistaken about itself.
2. Salience^3^: For a well-functioning group it seems absurd to avow uncertainty about the group’s attitude. If the group is successfully asked “Do you believe that p?” it seems absurd to answer “We don’t know.” However, it might not be absurd to answer that in the sense of suspending judgment. It would only be absurd if the group did not know whether they believe that p, whether they do not believe that p, or whether they are withholding judgment.^4^

For instance, a mission statement is a group avowal with which the group self-ascribes certain core beliefs, goals, and desires. Usually, such a mission statement comes with *weak authority*. A challenge of a mission statement is in most cases about its sincerity, or perhaps about the likelihood of achieving the goals within the mission statement. Only in very extraordinary circumstances is it appropriate to challenge whether a sincere group actually has the beliefs or intentions self-ascribed. Moreover, for a well-functioning group (with all the operative members present) *salience* also shows itself in example cases. When Apple’s board of directors is asked whether they plan to release a new iPhone, it would be absurd for them to answer “We don’t know” as an expression of genuine ignorance rather than suspension of judgment. It is appropriate for them to say that they do not know yet, or are in the process of finding out, but complete ignorance seems absurd.

One might be skeptical about the exact features identified here. Nevertheless, it is widely accepted that something in the vicinity of features (i) and (ii) should be explained by theories of self-knowledge in individuals^5^ and Schwengerer (2022) shows that the same reasoning applies to theories of collective self-knowledge and the features (1) and (2). However, there are three more clarifications required for Schwengerer’s features of group avowals.^6^

First, an important difference between individual avowals and group avowals is that group avowals miss a feature that individual avowals have: *immediacy*. Wright (1998, 2015) proposed that whenever individuals avow a propositional attitude, it seems inappropriate to respond by asking for evidence —or asking “How do you know?”. However, as Schwengerer (2022) argues, this is not necessarily the case for group avowals. It can be appropriate to ask groups how they know that they believe p and they can answer by citing internal discussions, the expertise of particular members, voting within the group, and so on. Moreover, it is not only in exceptional circumstances that the question “How do you know?” is appropriate. It seems to be a felicitous question in all the paradigmatic examples of authoritative group avowals. It is still a little odd to respond to Labour’s manifesto—a paradigmatic group avowal—by asking how they know that is what Labour intends. However, the oddness does not come from the question itself, but rather from the perceived obviousness of the answer. They discussed and voted on the contents of the manifesto. There is something to be learned from typical answers to questions such as “How do you know that you intend to such-and-such?” that I will come back to later.

Rejecting immediacy also indicates another difference between Schwengerer and Wright. Whereas Wright uses the features of avowals in part to distinguish between a special form of self-ascription and those self-ascriptions that are done based on self-interpretation, Schwengerer does not do so. He holds that an avowal can (at least in principle) be based on self-interpretation. This fits with his other work on individual self-knowledge that proposes an account close to self-interpretation (Schwengerer, 2019). An avowal so understood is merely a self-ascription that satisfies weak authority and salience—regardless of the underlying basis for the self-ascription. Self-interpretation can potentially lead to avowals.

Second—and somewhat related—Schwengerer’s features of group avowals still include a reference to “empirically assumptionless justification.” For Wright, this would naturally be read as being connected to the immediacy of avowals, but given that Schwengerer has dropped immediacy for group avowals, this either cannot be the proper interpretation here or Schwengerer should not have kept this part of Wright’s characterization. Unfortunately, as far as I can tell, there is little argument in Schwengerer (2022) for keeping this clause. My suggestion is that one takes self-ascriptions to be justified by default before knowing anything about others’ reliability in their self-ascriptions. This seems like a plausible empirical claim to me. Importantly, it is not meant to be read as anything like an a priori justification for other people’s self-ascriptions. Whether an empirical claim to a default treatment of avowals as justified is captured well by talk of “empirically assumptionless justification” is questionable. Perhaps Schwengerer (2022) should not have used those words after all. In any case, I do not think much depends on this clause in the definition of weak authority for group avowals.

Finally, the definition of salience for group avowals refers to the seeming absurdity for the group to answer “We don’t know.” if the group is successfully asked “Do you believe that p?”. A reviewer highlights that this might be in tension with any inferential approach to self-knowledge. The worry is that inferential approaches might explain why it is unlikely that the group cannot answer the question “Do you believe that p?,” but it being unlikely is not the same as it being absurd. My first response here is that it is not quite clear how strongly “absurd” is to be read in Schwengerer (2022). The characterization of salience is loosely based on Wright (1998), but Wright does not use “absurd” to characterize attitudinal avowals. Wright only uses it for phenomenal avowals. This is a first hint that perhaps “absurd” here is not meant to be read in any strong sense, but should be read as compatible with something being merely very unlikely. Moreover, for salience in group avowals, the absurdity is first introduced qualified as “seems absurd.” In the following sentences, this “seems” is not explicitly there anymore, but at least for my purpose it is best to hold on to it. Something can “seem absurd” in case it is very unlikely, even though it is not actually absurd. If these observations are correct, then the reference to any absurdity here ought to be understood as a mere appearance of absurdity or an indication that something perplexing or abnormal is going on. When a group answers “Do you believe that p?” with “We do not know,” some explanation regarding why the group cannot answer is called for. It is an odd response. I do not think anything more is required for the salience of group avowals.

For now, let me summarize the two desiderata from our linguistic practice of group avowals.

Desideratum 1: The account of collective self-knowledge has to explain why group avowals are weakly authoritative.

Desideratum 2: The account of collective self-knowledge has to explain why group avowals are salient.

My account will explain these desiderata by looking at how collective self-knowledge is generated, rather than at the linguistic processes of expressing such self-knowledge. I propose that features about the relevant belief-formation explain and justify our linguistic practice of group avowals. Self-ascriptions of a group generated in the way I suggest are more reliable than usual ascriptions from the outside. Moreover, the basis of such a self-ascription is usually available to the group, hence the avowal shows salience. This is the target for my proposal.

### Groups

Any account of collective self-knowledge has to fit with background assumptions about the nature of a social group that has that self-knowledge. I will not argue for these background assumptions I am working with, but I am upfront in making them explicit. First, I assume that social groups exist. In our ordinary language, we talk about groups; in our law, groups are recognized, and groups are the target of scientific study. It seems difficult to deny that groups exist at all. Those groups come in different shapes and sizes: small, short-lived groups, such as three people working together to push a car; medium-sized philosophy departments; and even large groups with complex structures, including corporations such as Amazon or Apple. All of these are groups in some sense. What exactly makes them a group is contested. Ritchie (2013) proposes that groups beyond mere feature groups realize a structure. Others propose that groups have some specific part-whole or grounding relations (Epstein, 2015; Hawley, 2017). Moreover, a tradition focusing on group action emphasizes the role of collective intentionality as the paradigmatic feature of groups. They discuss how groups bind their members together in virtue of some form of shared or joint intention (Bratman,^7^,^1993^, ^2014^; Searle, 1995, 2010; Tuomela, 2004, 2005, 2013 Gilbert, 1989, 1990; 2009; 2014; Pettit, 2001, 2007; List, 2005; List & Pettit, 2011; Schmid 2009). I aim to remain as neutral as possible about these positions. I only presuppose that social groups exist and are more than a mere collection of individuals.

Second, I also assume that groups can have attitudes that are more than just metaphorical attributions and more than merely the sum of the attitudes of individual group members. This assumption is usually labelled as *inflationary* or *non-summativist*. The whole idea of looking for collective self-knowledge would make little sense without such an assumption. Of course, that assumption is not uncontroversial (see for instance Quinton (1975) and Rupert (2005, 2019)), but it is not the aim of my paper to defend those attitudes as such. Plenty of arguments in favor of group attitudes have been given in the past. For instance, Tollefsen (2002a) and Kallestrup (2020) argue from an observation of ordinary linguistic and normative practice in which attitudes of groups play a role. Tollefsen (2002a, 2002b) and Huebner (2014) argue for a group’s attitudes based on an intentional stance (cf. Dennett (1987)). Theiner et al. (2010) and Lyre (2018) point to socially extended knowledge as a path to attitudes for groups. I will not provide any new arguments to add to this (incomplete) list. My starting point is the existence of inflationary, non-summativist group attitudes. Nevertheless, I remain neutral about the precise nature of these attitudes. My goal is to develop an account of collective self-knowledge that is compatible with as many accounts of group attitudes as possible. This is captured in my third desideratum:

Desideratum 3: The account of collective self-knowledge should be compatible with as many theories of group attitudes as possible.

The rationale for this desideratum is the following: within the camp of philosophers accepting the existence of group attitudes much is still under debate. The current landscape of that debate does not favor any particular view of group attitudes. As such, any account of collective self-knowledge that only functions with one particular theory of group attitude is on risky grounds. However, if there can be a structural account of collective self-knowledge that is compatible with many different theories of group attitudes, then that account can avoid those risks. I think such a neutral account is possible. I do not claim that this account captures the only way to gain collective self-knowledge, but it is one that we can safely work with.

This final desideratum is of a different kind than the first two. The first two can be fully satisfied, or not satisfied. The third desideratum is more of a gradual affair. It can be satisfied more or less.

## Towards inferential collective self-knowledge

Inferential accounts of self-knowledge start with a simple idea: one infers that one has a particular attitude from evidence. As such inferential accounts of self-knowledge are especially *economic*. An inferential account does not require us to postulate any unique faculties in our cognition that facilitate the acquisition of self-knowledge. The way one infers one’s attitude from evidence is no different than coming to know anything else by performing an inference from evidence. I can infer that it must have rained earlier from looking at the wet streets. Here the wet streets (or my representation of them) serve as evidence for my belief that it must have rained earlier. Inferential accounts of self-knowledge hold that a very similar inferential process is involved when we acquire self-knowledge. And because we already accept that we can make inferences, the proposal that the same ability is involved in generating self-knowledge is especially parsimonious. No additional abilities or faculties are required. Some alternative accounts of self-knowledge are far more demanding in that regard. For instance, inner-sense accounts (e.g., Armstrong (1968) or Goldman (2006)) require either a unique organ for an inner sense, or at least a cognitive ability or faculty realized in one’s brain that is dedicated to detecting one’s own attitudes. This economic nature is also what makes inferential accounts of self-knowledge attractive for an account of collective self-knowledge. Some accounts of groups and their attitudes reject anything like an organ or cognitive faculty of introspection available for social groups. They hold that a social group is realized in its members and their relations without any room for unique group organs for introspection. Inner-sense accounts seem a non-starter for collective self-knowledge for such conceptions of social groups. Of course, ruling out inner-sense accounts by itself is just eliminating one of many options for self-knowledge theories. But what the contrast to inner-sense accounts illustrates is how minimal the requirements for an inferential account are. All that we need is some evidence and a way to make inferences based on that evidence. And that groups can have evidence and can make inferences based on that evidence is already widely accepted. This is the virtue of inferential accounts.

One might worry here that this is a little quick. Even though most people accept that groups can have evidence, they are contesting the nature of that evidence. For instance, some might think a group’s evidence is just a matter of joint acceptance (Hakli, 2011; Schmitt, 1994; Schwengerer, 2023a, 2024), while others vehemently argue that it is not (Lackey, 2016, 2021). Fortunately, for my purpose, it does not matter much what the exact nature of group evidence is. All that I require is that the evidence a group has, or should have, is such that it allows for an inference to the group’s attitudes. Some joint acceptance accounts might not satisfy that criterion, but even among joint acceptance accounts, some versions anchor evidence the group has or should have sufficiently to the world.

Having decided on an inferential account, the central question is what evidence a group can use to infer its own attitudes—its own beliefs, desires, intentions, and so on. There are broadly two ways to go here. The first option is to look for evidence that indicates that the group has a certain attitude because the evidence is likely a result of that attitude. This is well-known from inferential accounts of self-knowledge in individuals. A person who responds to a spinach dish by refusing to eat the spinach likely dislikes the taste of spinach. The action of the person is evidence of a certain mental state of that person. In more elaborate forms this approach has been explored by Lawlor (2009) and Cassam (2014). It is an option for groups as well. At least sometimes groups can observe their own behavior and then infer what sorts of beliefs that behavior most likely indicates. However, it cannot be the usual path for collective self-knowledge. It jars with the ordinary linguistic practice of group avowals.

To see the problem of this first option it is helpful to look at the one missing feature of group avowals. Group avowals are not immediate. When a group is asked “How do you know that you intend to φ?” the group usually can give answers other than “We just know.” Let me illustrate this with an example. Suppose Microsoft avows by saying “We want to expand and buy more companies” at a press conference after a shareholder meeting. A member of the press asks “How do you know?”. In contrast to an individual avowing, a group can be asked this question appropriately. And the natural answer does not look anything like an inference from past behavior that is indicative of a desire. Microsoft will not say “We observed our recent actions, and they indicate that we want to expand and buy more companies.” The answer will rather be something akin to “We discussed whether we want to expand in the shareholder meeting and concluded it is the best choice of action to expand now” or “We discussed it at the meeting and the shareholders voted in favor of continued expansion and acquisition of promising smaller companies.” There is also a potential response that does include an observation of the company’s past, but crucially it uses that past not as an indication for having a certain attitude, but as (part of) the grounds for new decision-making. For instance, Microsoft might say “We did not expand aggressively enough in the past when we had good opportunities, so we decided to change our strategy and want to acquire more companies.” Here the past is considered in making a decision. Sometimes this is done explicitly, but some decisions can be based on past experiences in a way that is less obvious and results from the past inform current decisions in ways that need not be explicitly discussed within the group.^8^

All these responses point to the second option for inferential accounts of self-knowledge: the basis for the inference is the behavior that likely *causes* the attitude, not the behavior that is likely *caused by* the attitude. Taking this second option, inferential self-knowledge is a form of reliably predicting what attitude a group will most likely have at t_1_, given the known states and processes within the group at t_0_. In the Microsoft case, the group observes the shareholder meeting, the discussion within that meeting, and most importantly the voting on a decision afterwards. The group can then infer based on those observations what the group wants or desires.^9^ If the voting is in favor of continued expansion and buying other companies, then the group most likely now has a desire for continued expansion and buying other companies. The process that determines the group’s desire is also the inferential basis for the self-ascription of that desire. The inference needs to go forward from evidence about the causes to the caused group attitude. This is the option I want to build on.

## Looking back and looking forward—the account

The two options differ in the causal direction of the evidence considered. In option one, the evidence is used to infer the causes of that evidence. A group observes its own behavior^10^ and uses the observations to infer the cause of the behavior. Microsoft behaves in a particular way because they want to expand, and that desire to expand causes the group to behave like that. The group looks at events that are caused by the group’s attitude to then infer what that attitude is. The inference goes backwards: from the caused events to the causing attitude.

However, as shown above, the inferential account of collective self-knowledge of attitudes needs to look at evidence in a different place. The account should not focus on events that are caused by the attitudes, but on events that cause the attitude. The inference needs to go forward as suggested in option two.^11^ This is not to say that the inference always has to go this direction, but rather that it seems promising to explore an account in which the usual inference goes forward rather than backwards.

With this in mind, I can look at paradigmatic collective self-knowledge of attitudes. For those paradigmatic cases, the evidence basis for an inference has to be about *the process of forming that attitude within the group*. Whatever that process of forming a group’s attitude is, any observation or representation of that process constitutes the evidence that allows the group to infer the attitude. The sort of inference in play is akin to an informed prediction: given the group and its structure, what attitude would most likely be formed by the group’s attitude-forming process at time t?

The details of these pictures have to be filled in by considering the background views one accepts about group attitudes. Depending on how groups form attitudes in the respective account, the evidence will look slightly different. This makes a very flexible account of collective self-knowledge for attitudes.

Let me illustrate the general idea with a case of group belief in Gilbert’s (1989) joint commitment account. Suppose a group forms a group belief^12^ by establishing a joint commitment that is expressed openly. This mechanism involves some form of communication that establishes the group belief. Person A expresses their willingness to jointly commit to p. Person B expresses their willingness to jointly commit to p. Person C,… and so on. When every involved person expressed their willingness in a setting with common knowledge, a joint belief that p is established for the group. This mechanism bringing about the joint belief via joint commitment can be used as evidence by the group to infer what attitude the group is about to form. If the group members are about to form a group belief by expressing a willingness to commit themselves to p as the group’s belief, then the group can use those expressions of willingness as a basis to infer that the group believes that p. If the group is asked “How do you know that you believe that p?” the group can answer “We talked and each of us openly expressed that they are willing to believe that p as a group.” Or perhaps merely “We discussed the issue and agreed that p”.

The same approach also works if one accepts a judgment aggregation perspective as the background theory. Suppose a group forms the belief that p by premise-based majority voting similar to the suggestion in List & Pettit (2011). Here the group belief is generated by looking at the individual judgments of the premises.

Suppose we have a three-person group that deliberates on whether the group believes that q, based on considerations of p and p → q. Hence, in this scenario, the propositions p and p → q are designated as premises and the proposition q as the conclusion. The profile of the individual’s judgments and the majority judgment is as follows (Table 1):

**Table 1:** Belief aggregation with List & Pettit

	**p**	**p** → **q**	**q**
Person 1	True	True	True
Person 2	True	False	False
Person 3	False	True	False
Group	True	True	True

Individually, only Person 1 believes that q. Person 2 and Person 3 do not believe that q because they either reject p → q or p. However, because the group uses premise-based majority voting as their judgment aggregation method, the group only votes on p and p → q, not on q directly. The majority votes in favor of p and also in favor of p → q. As a result, the group then infers from the accepted propositions that q is also true.

My proposed account of collective self-knowledge of attitudes uses the judgment aggregation method and concrete voting within the group as evidence. The group infers what the group believes based on (1) the general premise-based majority voting practice, (2) the voting results about proposition p, (3) the voting results about proposition p → q, and (4) the group reasoning that provides the inference to q.

This fits exactly the answer we expect if we ask the group “How do you know that you believe that q?”: we know because the group voted on the premises and then made an inference based on the results. (1) to (4) are all observable internal behavior that constitutes the evidence. The group merely needs to take that evidence and infer what the group most likely believes, given that evidence. And in the case of premise-based judgment aggregation, it is very easy for the group to infer what they believe, based on that evidence. It is almost impossible to fail that inference.

Of course, the account still has to be developed in detail in the List & Pettit framework. I can only sketch which path such a development would likely take. To capture (1) to (4) properly in List & Pettit’s premise-based majority aggregation framework,^13^ (1) to (4) have to become premises for the group so that the inferences can be drawn. Making a proposition a premise is a somewhat arbitrary decision that the group needs to make in order to avoid the irrationality of having conflicting beliefs. For my purpose, (1) to (4) would need to become premises, because they are supposed to be the basis for the inference. Given that this approach also uses majority voting to determine group beliefs, (1) would be the group belief determined by the group members’ votes on whether the group functions with a premise-based majority voting practice; (2) would be the group belief determined by the group members’ votes about the voting results regarding p; (3) would be the group belief determined by the group members’ votes about the voting results regarding p → q; and (4) would be the group belief determined by the group members’ votes regarding how the group reasons (e.g., that the group is rational and which logical rules the group follows). The resultant group beliefs that are used as premises are primarily about the belief-aggregation of the group itself. This is expected for a path to self-knowledge, as the group takes on a meta-perspective when looking at its own attitudes. Going through the propositions in question 1 would end up with a very large and complex table that includes enough for the group to infer that the group believes q (likely including some intermediary steps with which the group infers that the group believes p and p → q). The inference the group then performs looks roughly like this (with the premises being established by majority voting):

1. Group beliefs are determined by a premise-based majority voting practice. [Premise]
2. The majority voted that p is true. [Premise]
3. The majority voted that p → q is true. [Premise]
4. The group follows logical rules of reasoning including Modus Ponens. [Premise]
5. The group believes p. [From 1, 2]
6. The group believes p → q. [From 1, 3]
7. The group inferred q from p and p → q [From 4, 5, 6]
8. The group believes q [From 4, 7]

This sketch of how the account can be further developed in List & Pettit’s framework is very tentative and still incomplete.^14^ It also currently ignores many possibilities of distributing epistemic labour that are available as further refined ways to aggregate judgments. Nevertheless, I hope it makes the path the group takes in inferring their belief more concrete.

For both these examples—joint commitment and premise-based judgment aggregation—the account of collective self-knowledge of attitudes is indirect. The attitude of the group is not directly accessed. Instead, the group members observe the group’s attitude-forming process and use that observation as evidence for the group to infer the generated attitude. This ensures that the account stays economic. No special faculty of group introspection is required. All evidence is behavioral and accessible to the group members who then can reason and perform the required inferences either jointly or by distributing epistemic labour over the group members.

## Access to evidence and basing

My proposed account so far explains what evidence is used in paradigmatic cases of collective self-knowledge. The group accesses the process that forms the group’s attitude and then uses that process as evidence. Importantly, the access a group has to the process of forming a group’s attitude depends on the details of the background theory of group attitudes one accepts. The theory of group attitudes gives us the process in which these group attitudes are formed and depending on that process we can determine the group’s access to the evidence used to acquire self-knowledge. For instance, if one accepts Gilbert’s joint commitment account one also accepts a common knowledge condition on each involved person’s willingness to believe p as a group. And this common knowledge constitutes access for the group via its members to the evidence that can be used to infer that the group believes that p.

Other background theories of group attitudes lead to different access relations to the group’s attitude-forming process and hence to the evidence paradigmatically used for collective self-knowledge. Some theories give us transparent formation of the group’s attitudes—the formation occurs under common knowledge, or with every group member in a position to know how the group forms an attitude. However, some theories might involve an attitude formation in the group that is rather opaque. A theory might hold that group attitudes supervene on individual group member attitudes in a way that usually cannot be recognized by the group or its members. In such a case, there would be very little evidence that could be used for collective self-knowledge. In general, the better the access to the group’s attitude formation, the better the evidence available for the group and the more likely the group self-ascribes an attitude correctly based on that evidence. This also entails that groups should be structured in a way that fosters transparent attitude formation in the group in order to support collective self-knowledge.

I have mentioned a group’s access to evidence via its members. At this point, I can distinguish between the evidence that group members have and the evidence the group has. Under some accounts of group evidence, they will be very close, if not the same. For instance, one might accept a pooling account in which the group’s evidence is constituted by all the evidence the group members have (for a discussion of pooled evidence see Brown (2022b)). The other extreme takes the group evidence to be completely (or mostly) independent of group member evidence, such as joint acceptance accounts of group evidence (Hakli, 2011; Schmitt, 1994; Schwengerer, 2023a). A group’s access to the evidence that can be used to acquire self-knowledge will also depend on the relation of group evidence to group member evidence. Take again Gilbert’s joint commitment account. Here the group members have evidence about the group’s belief-forming process. This member evidence then needs to become the group’s evidence (e.g., by another joint commitment). Only then the group itself can infer its attitude based on that evidence.

Of course, even if a group has the required evidence at a group level, this is not enough for collective self-knowledge on its own. The group also needs to form the second-order belief that the group believes that p properly based on that evidence. I will not give a detailed account of basing in this paper. All that is required here is a simple, causal account of basing as proposed by Brown (2022a). The evidence has to cause the second-order belief. In my account, it has to cause the second-order belief via an inferential process.

## Meeting the desiderata

The proposed account can meet the desiderata. First, it explains why group avowals are weakly authoritative—it explains Desideratum 1:

Desideratum 1: The account of collective self-knowledge has to explain why group avowals are weakly authoritative.

Group avowals are rarely doubted because they are based on evidence that allows for an especially reliable belief-forming process. Because the attitude-forming process itself is used as evidence for self-ascribing the attitude, the inference is reliable. And because the inference is reliable, the resultant second-order belief is likely true and the avowal is authoritative. Generally, the reliability of the inference is explained by the fact that the basis for the inference is constitutive for the group attitude that is self-ascribed. The attitude-forming process includes the supervenience basis for the group attitude. Hence, if the group can access the attitude-forming process—that supervenience basis—as evidence, it is rather easy to make a good inference to what attitude the group most likely has. For instance, in List & Pettit’s premise-based majority model, a group belief that p can be constituted by the voting procedure and the particular votes. Because of that constitutive relation between the voting and the thereby established group belief, it is easy for the group to infer the group belief on the basis of the voting. However, because it is an inference, there is no guarantee that the inference is done correctly. Hence, challenging the avowal is possible, and it is merely *weakly* authoritative. A legitimate challenge demands evidence that the group failed the inference or used insufficient or misleading evidence. The degree to which a group can fail in these ways depends on the background account of group attitudes one chooses. For instance, in List & Pettit’s framework, it will be very difficult to provide evidence for any failure of inference. Suppose a group follows a premise-based majority model with voting and the majority of the members believe that p and vote accordingly. Following the majority, the group also believes that p. Given my proposal, the group can now infer that the group believes that p, by looking at the member beliefs (e.g., the votes) and the majority-based aggregation function. Given those, the group self-ascribes that it believes p. It might seem almost impossible to have gone wrong here. Nevertheless, mistakes in making inferences are not ruled out. Even in the simplest inferences one might be too hasty or careless and get things wrong. One can even make mistakes when performing a simple Modus Ponens. It is very unlikely, but not impossible for non-ideal groups. Hence, to legitimately challenge a group avowal in the List & Pettit framework, strong evidence of the group’s failure in performing a proper inference will be required.^15^ Other background accounts can lead to the group incorrectly self-ascribing an attitude more easily. In those cases, challenging the group avowal will also be easier.

Desideratum 1 requires not merely reliability, but rather a comparative advantage in reliability between the group self-ascribing an attitude and outsiders ascribing an attitude to the group. For most background views of group attitudes, the evidence used in the inference to self-knowledge in my account does not constitute such a comparative advantage. Whoever has the evidence can make a reliable inference. However, a group’s advantage comes from better access to the evidence in ordinary circumstances. In the Gilbert model, the group has a particularly good access to the group member’s willingness to jointly commit to something. In the List & Pettit model, the group is in a particularly good position to know the voting practice and results. Outsiders can in principle have equally good access, but usually do not have that. My explanation for weak authority therefore relies on an empirical claim that groups are generally in a better position to observe their attitude-forming process than outsiders are. This leaves room for exceptions but provides enough comparative advantage in the reliability of self-ascriptions to establish weak authority.

A similar explanation that relies on the availability of evidence for the group can be applied to Desideratum 2:

Desideratum 2: The account of collective self-knowledge has to explain why group avowals are salient.

Salience holds that if the question “Do you believe that p?” (or corresponding questions for other attitudes) reaches the group, it appears inappropriate—perhaps even absurd—for the group to answer “We do not know.” My account explains this observation by the availability of the relevant evidence for the group. If the attitude-forming process is easily available as evidence for the group, then Salience follows naturally. For many accounts of group attitudes, this easy access is accepted. Both my examples, Gilbert’s (1989) joint commitment account and the premise-based majority voting in List & Pettit (2011), are naturally described as including such easy access. Gilbert has the common knowledge condition built-in and premise-based majority voting functions with the vote results being known by the group members. These features allow the group to access the attitude-forming process as evidence for self-knowledge.

Both answers to Desiderata 1 and 2 rely on features of the background accounts of group attitudes. The account gives the right results only if the background account allows for the evidence to be easily accessed by the group. Hence, there is a tension between satisfying Desiderata 1 and 2, and Desideratum 3:

Desideratum 3: The account of collective self-knowledge should be compatible with as many theories of group attitudes as possible.

Can desideratum 3 nevertheless be satisfied? The desideratum demands compatibility with as many theories as possible. However, it would be unreasonable to demand that the proposed account is compatible with every background theory of group attitudes. Compatibility with more background theories is a virtue of a theory, but as long as there is no other account that is compatible with more background theories and still able to satisfy desiderata 1 and 2, my proposal is still the best account on offer.

Moreover, even though the satisfaction of desiderata 1 and 2 in my account demands background theories for attitudes that have certain features, these features are widespread in the landscape of theories of group attitudes. Many different theories allow for easy access to attitude-forming processes in groups. All theories that include a common knowledge condition for group members are well suited. This includes Gilbert (1989, 1990; 2009; 2014), Tuomela (2004, 2005, 2013), and Bratman (1993, 2014). All theories in which forming a group attitude involves some form of voting that is observable (e.g., List & Pettit (2011)) are well suited too. On the other hand, it is less clear whether interpretationist accounts such as Tollefsen’s (2002a) fit. One might worry that interpretationist accounts do not have easily identifiable processes of attitude generation that can be used as evidence and hence my account is more limited than desired. I could concede this and nevertheless hold that my account functions for many, though not all theories of group attitudes. However, I am going to show that even Tollefsen’s background theory can be combined with my approach to collective self-knowledge. Hence, I will end my discussion by taking on the challenge of illustrating an interpretationist version of my proposal.

## A challenge: self-knowing groups and interpretationism

Tollefsen (2002a) proposes an interpretationist model for group attitudes. Roughly, interpretationism is the view that if an agent can be successfully interpreted as having an attitude A, then that is sufficient for that agent being intentional and having attitude A. Successful interpretation justifies the ascription of such an attitude. The ascribed attitude explains the agent and their actions. This explanation functions by treating the agent as a rational being who acts for reasons. It makes the agent intelligible to us by ascribing the same principles of rationality to the agent that we take to hold for ourselves. Tollefsen gives us an incomplete list to illustrate these principles: “do not act contrary to your best judgment, draw inductive inferences on the basis of all available, relevant evidence, believe only things you take to be true, and don’t believe inconsistencies” (Tollefsen, 2002a, p. 399). Interpreting agents works by assuming the agent follows these principles and then figuring out what attitudes the agent most likely has, given both their behavior and those principles. For instance, an agent who brings an umbrella can be interpreted as believing that there is a good chance of rain. That would be a reason why a rational being following their evidence and best judgment would bring an umbrella.

Tollefsen follows Dennett in that having a particular attitude is nothing other than being an intentional system that can be successfully predicted by ascribing that attitude in this way. In Dennett’s words: “all there is to really and truly believing that p (for any proposition p) is being an intentional system for which p occurs as a belief in the best (most predictive) interpretation” (Dennett, 1981, p. 68). Importantly, being an intentional system in this sense is not limited to human beings. Groups can be interpreted in the same way as individual agents. One can ascribe a rational point of view to a group and make sense of the group’s actions by invoking the same sort of rationality principles used in interpreting individuals. Tollefsen illustrates this with Ford Motors:

What will Ford Motor Company do in response to the rise in gas prices? If Ford is rational then it will act so as to maximize its profits. Because Ford wants to avoid losing money on its line of large vehicles and believes that individuals are less likely to buy large vehicles during a time at which gas prices are high, we can predict that Ford will discount its large vehicles. (Tollefsen, 2002a, p. 402)

Tollefsen shows that one can happily interpret Ford Motor Company as having particular wants and beliefs that are related by rationality principles. And there is nothing more to a group having wants or beliefs than being interpreted in this way. The interpretation captures a “real pattern” (Dennett, 1991) in Ford’s behavior and reasoning that can be simplified with the ascription of beliefs, wants, and desires.

The challenge for me is now to find a way to combine my proposal of collective self-knowledge with this interpretationist picture of group attitudes. The difficulty here is that in the interpretationist story about Ford, we do not have a process that generates an attitude that is explicitly described in non-attitudinal terms. In Gilbert, we have observable expressions of willingness to jointly commit. In List and Pettit, we have an observable voting practice that determines a group attitude. But in Tollefsen’s picture, there is no single observable path to group attitudes. There is no easy way to identify an attitude-forming process for the group. As such, the interpretationist challenge is exemplary for broadly functionalist views of group attitudes in general.

To apply my proposal of collective self-knowledge to Tollefsen’s view, I need to start with the basic idea that explains the reliability of self-ascriptions in my account. In the Gilbert framework, the joint commitment is good evidence for ascribing a belief to the group because the joint commitment establishes that group belief. In the List and Pettit framework, the voting is good evidence for the same reason: it establishes the group belief. For an interpretationist framework, I therefore also need to look for whatever establishes group attitudes. If a group belief is nothing but a real pattern in behavior and reasoning of the group, then I can identify the group belief with a complex set of dispositions that accounts for that pattern. Then the relevant question for applying my account is the following: what is the origin of this complex set of dispositions? What grounds the real pattern? The natural interpretationist answer is to point to the individual group members and the structural features between them. The group’s dispositions are grounded in the member dispositions and the way the members are related to each other. This parallels the interpretationist framework for attitudes in individuals which are also real patterns that are grounded in dispositions on a lower level of analysis—roughly dispositions of the parts our body is made from (cells, enzymes, hormones, etc.…) and their relations to each other. The dispositions of the more complex thing are grounded in the dispositions of its parts and the structure they are organized in.

If this model of group attitudes as real patterns grounded in individual dispositions and structural features is right, then this ground can play the same evidential role for an inference to self-knowledge that joint commitments play in a Gilbert framework, or the votes play with a List and Pettit background. Individual dispositions and the structural relations between individuals can constitute the evidence from which the group can infer its own attitudes. And such an inference can be especially reliable because that evidence has the right sort of grounding relations to group attitudes. If the group has sufficient knowledge of those grounding features, the group can very reliably infer the group attitude. In this way, my account works for interprationism the same way as it works for Gilbert or List and Pettit background views.

A natural objection here is to wonder why the group should even bother with dispositions of individuals and structural features of the group. After all, the interpretationist might say that what the real patterns are grounded in does not matter, as long as we can interpret the group as having certain attitudes. Looking at the lower level is a lot of work for little gain, so the group should just stick with self-interpretation on the higher level.^16^ Here, I respond with two points: First, detecting factors that ground group attitudes need not be very difficult. Sometimes the dispositions of individual group members are easy to see, and it takes little work to use them to infer the group attitude. Individual members can even tell the group about them and thereby make that evidence available to the group. This is an important difference between the group case and the individual case. For an individual, it is usually difficult to detect whatever grounds their attitude. For a group, it need not be. Hence, it might not be so much work after all. Second, behavioral evidence of the group can at times be sparse and insufficient to make a good judgment about the group’s attitudes. In those cases, the interpretationist response is to ascribe what is most likely—what makes most sense to ascribe to the rational agent. But “most likely” given limited evidence is not the best thing to work with—especially when the target of interpretation is the interpreting group itself. The group needs good access to their own attitudes for their decision-making. Without identifying what the group wants, it cannot make good decisions. Therefore, it seems wise for the group to use any and all evidence it can access to self-ascribe attitudes. It is the rational thing to do.

What I end up with is twofold: first, evidence based on grounding factors can be relatively easy to acquire for the group; and second, the usual interpretationist route can be at times difficult for self-ascriptions of attitudes by the group. Hence, even within the interpretationist framework, the group should (also) use the easily accessible evidence of grounding factors and use them to self-ascribe attitudes when needed.

Nevertheless, one more caveat is needed: Even though I emphasized the general ease of accessing the dispositions of the group members and the structure of the group, there are also times when these dispositions and the group structure are rather opaque. In those cases, the group’s access to the relevant evidence is limited. This brings the authority of group avowals in question. Fortunately, I am only committed to a weak authority claim and I concede that authority comes in degrees. All that I need are reasons for why a group is usually in a better position to ascribe its own attitudes than anyone from the outside. This can be achieved even in the interpretationist picture. The group is usually in a particularly good position to find out about the group member dispositions and the group’s structure. This particularly good position is established insofar as the group members themselves are in a privileged position to know about their own dispositions and—usually—can testify to those dispositions to the rest of the group.^17^ Moreover, the group also has particularly good access to the group’s structure: either because the group explicitly determines its own structure in formal groups or because the interactions between group members can make the structure salient to those members in more informal groups. Granted, there might be exceptions in which group structures are (sometimes intentionally) hidden from the members, but all that I require is that by and large groups have good access to their own structure and the member dispositions. Weak authority of group avowals does not require exceptionless privileged access for groups. As long as usual, paradigmatic groups have better access to the evidence that allows the group to infer their own attitude weak authority can be explained.

Moreover, that access to the relevant evidence comes in degrees is an advantage rather than a problem. It helps me to explain why some groups easily acquire self-knowledge, while other groups struggle. It also allows to include the ease of acquiring self-knowledge in the design of structured groups. We can design groups and their decision-making processes in ways that allow for easy self-knowledge. And we can identify groups that might be designed to make self-knowledge as difficult as possible on purpose. For instance, it is conceivable that one could intentionally form a political group that works against the interests of both the group itself and its members, and structure the group in a way that obfuscates those goals. A foreign intelligence could create or support a political party that aims to destabilize the current political system—including the party itself—without the party recognizing that aim. My account leaves at least theoretical room for such groups—especially when combined with an interpretationist (or some other functionalist) background account of group attitudes.

Overall, even though applying my proposed account to Tollefsen’s framework is less straightforward than applying it to Gilbert’s position, or List and Pettit’s view, I can nevertheless make it work. This speaks further in favor of the account satisfying desideratum 3.

## Collective self-ignorance and collective self-deception

I want to end with some remarks on the failure of collective self-knowledge.^18^ The process of generating self-knowledge in individuals is fallible and so is the one in groups. How easily it can fail depends on the background account of group attitudes of one’s choice. But any account will include a possibility for groups to fail in self-ascribing an attitude correctly if it is done based on an inference. Any process that involves an inference has some risk of failing in performing that inference—even very easy inferences based on good evidence. Sometimes groups can also fail to gain self-knowledge by an inference because they do not recognize all the relevant and available evidence, or because the relevant evidence is not available anymore. Take one example for the latter case: Suppose a group has decided whether they want to φ by jointly deliberating on the advantages and disadvantages of φ-ing (where φ-ing is some long-term activity). The next day, the group meets again, but some members who usually remember the group decision-making very well are missing. The group then wonders whether they want to φ, but because the deliberation happened yesterday and the members with great memory of the deliberation are missing, the currently active group does not know whether the group wants to φ. The relevant evidence is currently unavailable to the group, but the group plausibly still wants to φ. They just don’t know it.^19^ As soon as the other members with their better memory are present again, the group will quickly realize that they want to φ by considering yesterday’s deliberation^20^ and then act accordingly.

Moreover, groups can also refrain from performing any process that would lead to self-knowledge at all. This could be because the group decided not to look into their attitudes, or because the question of looking into it was never considered in the first place. Both an incorrect self-ascription, and the absence of a self-ascription can constitute a case of self-ignorance. I understand self-ignorance as a mere lack of knowing the group’s attitude.^21^ This has to be distinguished from self-deception cases, which also feature a lack of knowledge of the group’s attitude, but include some motivational component that leads the group to be self-ignorant. Whereas the self-ignorant merely fails to know their attitude, the self-deceptive also wants to fail to know their attitude. Groups can be both self-ignorant and self-deceptive.

Given that I understand self-ignorance and self-deception based on the lack of self-knowledge, much of what applies to group knowledge also applies to self-ignorance and self-deception. Group knowledge—like any group attitude—is understood in an inflationist way. Hence, the lack of such knowledge is also understood inflationist. A group does not necessarily lack knowledge just because a single member lacks knowledge. For collective self-knowledge, that entails that there will be cases in which some group members are ignorant about a group attitude, but the group nevertheless has self-knowledge of that attitude. Which and how many members can be ignorant while the group still has self-knowledge is a tricky question and largely depends on the background account one works with. Moreover, it will depend on the particular structure of the group in question. As a general rule, only members who are in some way involved in decision-making (operative members) will matter with regard to collective self-ignorance. Group members who do not influence the group’s beliefs about the group’s attitudes clearly cannot make the group self-ignorant. A low-level Apple salesman plausibly does not influence Apple’s decision-making at all. Hence, if they do not know whether Apple wants to expand into new markets, that will not affect Apple’s self-knowledge about that want. On the other hand, if Tim Cook is ignorant of strategic goals and desires of Apple, that will likely influence Apple’s group belief about these goals and desires and could lead to collective self-ignorance.

Interestingly, all of these failures to know the group’s attitudes can be detrimental to Apple. When any member of Apple fails to know Apple’s attitudes, they might have a hard time aligning their actions with Apple’s goals (cf. Schwengerer (2025), Boswell (2006), Boswell & Boudreau (2001), Boswell et al. (2006)). This goes for both the low-level salesman and Tim Cook. Of course, the consequences will be more severe in the latter case. In addition, Apple’s failure to know its own attitudes might be detrimental beyond the individuals failing to align their actions. It constitutes a misalignment between Apple and how Apple views itself. Apple might use their incorrect self-ascription in decision-making and end up acting against their own desires. And it might take the group a while to notice that they were wrong about their own desires. For Apple, that would be costly.

## Conclusion

I have argued for an original, inferential account of collective self-knowledge. Groups know their own attitudes by inferential means. They have a defeasible, privileged access to their own attitudes. The basis for inferring those attitudes is primarily the attitude-forming process that the group uses to determine their group attitudes, which accounts for the privilege in question. This is not true for every background view of group attitudes. But with most background views, the group has easy access to their attitude-forming process. I demonstrated this with three prominent accounts: Gilbert’s joint commitment account, List & Pettit’s premise-based majority voting, and the more complicated interpretationism by Tollefsen. I provided a first attempt to establish the explanatory power of my account. I furthermore showed how it can meet the desiderata sufficiently. The account can explain the features of group avowals, and it seems to be compatible with a vast majority of background views. Applying my account to other background views still needs to be done in the future to fully make good on the promise of an account that can be combined with as many background views as possible. Nevertheless, it is clear to see that the account has structural features that can be combined with almost any view of how group attitudes are constituted and generated. I have shown that even interpretationist views can be combined with my proposal. This provides a fruitful ground for further research.

## Data Availability

This paper does not analyze or generate any datasets.

## Footnotes

1 E.g. attitudes towards an object rather than a proposition.

2 Wright is interested in the speech act of self-ascribing mental states generally. I am limiting myself to attitudes.

3 Schwengerer (2022) speaks of “weak salience” because he holds that salience in groups is weaker than in individuals. The suggestion is that for groups, there can be cases in which someone asks the group “Do you believe p?” while the group is not in a state of being able to receive or answer the question properly. For instance, relevant group members might be missing when the question is asked. Hence, salience for groups includes a clause that refers to the group being “successfully asked” and being “well-functioning.” These qualifiers are meant to rule out cases in which relevant members are missing; or cases in which the group has not considered a proposition and is not in a position to do so currently. I am not convinced that there is such a big difference. There can be instances in which individuals are asked “Do you believe that p?” when not in a state of being able to receive or answer the question properly as well. Perhaps such cases are more common for groups, but the difference is one in degree at most. Hence, I speak of salience instead of weak salience.

4 I take withholding judgment here to include cases in which the group does not care whether p and hence does not bother to form a belief about p.

5 This can be seen for instance in Armstrong (1968), Burge (1996), Moran (2001), Nichols & Stich (2003), Byrne (2005; 2018), Goldman (2006), Boyle (2009; 2011), Fernández (2013) and Schwengerer (2019). They all hold that the nature and production of self-knowledge explain the linguistic features of avowals.

6 Thank you to a reviewer for prompting more detailed clarifications here.

7 Note that Bratman holds that shared intention can be analyzed in terms of individual intentions that are interrelated in the right way.

8 Thank you to a reviewer for pointing out this use of the past in decision-making.

9 In other cases, the inferential basis might be weaker. It is easy to infer the group’s belief from a vote, but not all group beliefs are best understood as being based on votes.

10 And in a more elaborate version also prompts some of that behavior intentionally to generate evidence. See Lawlor (2009), Cassam (2014) and Schwengerer (2023b).

11 The idea of a forward-looking inferential basis for self-knowledge is underexplored for individual self-knowledge. To my knowledge, the only account that builds on a somewhat similar idea is the predictive processing model in Schwengerer (2019).

12 I bracket criticism of Gilbert’s view that denies that joint commitment leads to mere acceptance rather than belief. See for instance Wray (2001).

13 This is not the only option for List & Pettit.

14 For instance, a more detailed account might need to provide even more motivation for accepting step 7, because groups might believe p and p→q without ever making the inference to q. As is, I assume that the group following rules of reasoning includes that the group makes these inferences, but a more careful way of spelling it out would include more conditions that capture that the group has considered q and that when the group considers q and believes both p and p→q, then the group performs the inference to q. Including this would have made the example even more unwieldy than it already is, hence I have simplified it a little.

15 Thank you to a reviewer for pushing for further clarifications.

16 Thank you to a reviewer for emphasizing this issue.

17 Usually, they testify not in terms of dispositions, but in terms of informing about their own individual mental states which are shortcuts for real patterns of individual behavior and reasoning.

18 Thank you to a reviewer for prompting this brief discussion.

19 Of course, writing it down would have helped.

20 Importantly, they will not re-decide to φ—they will recognize that they already wanted to φ.

21 “A lack of knowledge” is one common understanding of ignorance in general, although one that is contested. Proponents of this “standard view of ignorance” include Williamson (2000) and Le Morvan (2011; 2012). For an alternative and further discussion, see Peels (2010; 2023).
